# Supplementary material for: Resolving the full spectrum of human genome variation using Linked-Reads
Source: Genome Res. 2019 Apr;29(4):635–45. doi: 10.1101/gr.234443.118 (PMC6442396; doi:10.1101/gr.234443.118)
Supplement: Supplemental Material [file supp_gr.234443.118_Supplemental_longranger-2.2.zip › longranger-2.2.tar.bz2!/lr2.2-public/lib/stan/cmdstan/stan/doc/api/html/agrad_2fwd_2functions_8hpp.html]

Stan: src/stan/agrad/fwd/functions.hpp File Reference


|  |  |
| --- | --- |
|  | Stan  2.5.0  probability, sampling & optimization |


- Main Page
- Related Pages
- Namespaces
- Classes
- Files

- File List
- File Members

All Classes Namespaces Files Functions Variables Typedefs Enumerations Enumerator Friends Macros Pages

- src
- stan
- agrad
- fwd

functions.hpp File Reference

`#include <stan/agrad/fwd/functions/Phi.hpp>`  
`#include <stan/agrad/fwd/functions/abs.hpp>`  
`#include <stan/agrad/fwd/functions/acos.hpp>`  
`#include <stan/agrad/fwd/functions/acosh.hpp>`  
`#include <stan/agrad/fwd/functions/asin.hpp>`  
`#include <stan/agrad/fwd/functions/asinh.hpp>`  
`#include <stan/agrad/fwd/functions/atan.hpp>`  
`#include <stan/agrad/fwd/functions/atan2.hpp>`  
`#include <stan/agrad/fwd/functions/atanh.hpp>`  
`#include <stan/agrad/fwd/functions/bessel_first_kind.hpp>`  
`#include <stan/agrad/fwd/functions/bessel_second_kind.hpp>`  
`#include <stan/agrad/fwd/functions/binary_log_loss.hpp>`  
`#include <stan/agrad/fwd/functions/binomial_coefficient_log.hpp>`  
`#include <stan/agrad/fwd/functions/cbrt.hpp>`  
`#include <stan/agrad/fwd/functions/ceil.hpp>`  
`#include <stan/agrad/fwd/functions/cos.hpp>`  
`#include <stan/agrad/fwd/functions/cosh.hpp>`  
`#include <stan/agrad/fwd/functions/digamma.hpp>`  
`#include <stan/agrad/fwd/functions/erf.hpp>`  
`#include <stan/agrad/fwd/functions/erfc.hpp>`  
`#include <stan/agrad/fwd/functions/exp.hpp>`  
`#include <stan/agrad/fwd/functions/exp2.hpp>`  
`#include <stan/agrad/fwd/functions/expm1.hpp>`  
`#include <stan/agrad/fwd/functions/fabs.hpp>`  
`#include <stan/agrad/fwd/functions/falling_factorial.hpp>`  
`#include <stan/agrad/fwd/functions/fdim.hpp>`  
`#include <stan/agrad/fwd/functions/floor.hpp>`  
`#include <stan/agrad/fwd/functions/fma.hpp>`  
`#include <stan/agrad/fwd/functions/fmax.hpp>`  
`#include <stan/agrad/fwd/functions/fmin.hpp>`  
`#include <stan/agrad/fwd/functions/fmod.hpp>`  
`#include <stan/agrad/fwd/functions/gamma_p.hpp>`  
`#include <stan/agrad/fwd/functions/gamma_q.hpp>`  
`#include <stan/agrad/fwd/functions/hypot.hpp>`  
`#include <stan/agrad/fwd/functions/inv.hpp>`  
`#include <stan/agrad/fwd/functions/inv_cloglog.hpp>`  
`#include <stan/agrad/fwd/functions/inv_logit.hpp>`  
`#include <stan/agrad/fwd/functions/inv_sqrt.hpp>`  
`#include <stan/agrad/fwd/functions/inv_square.hpp>`  
`#include <stan/agrad/fwd/functions/is_inf.hpp>`  
`#include <stan/agrad/fwd/functions/is_nan.hpp>`  
`#include <stan/agrad/fwd/functions/lbeta.hpp>`  
`#include <stan/agrad/fwd/functions/lgamma.hpp>`  
`#include <stan/agrad/fwd/functions/lmgamma.hpp>`  
`#include <stan/agrad/fwd/functions/log.hpp>`  
`#include <stan/agrad/fwd/functions/log10.hpp>`  
`#include <stan/agrad/fwd/functions/log1m.hpp>`  
`#include <stan/agrad/fwd/functions/log1m_exp.hpp>`  
`#include <stan/agrad/fwd/functions/log1m_inv_logit.hpp>`  
`#include <stan/agrad/fwd/functions/log1p.hpp>`  
`#include <stan/agrad/fwd/functions/log1p_exp.hpp>`  
`#include <stan/agrad/fwd/functions/log2.hpp>`  
`#include <stan/agrad/fwd/functions/log_diff_exp.hpp>`  
`#include <stan/agrad/fwd/functions/log_falling_factorial.hpp>`  
`#include <stan/agrad/fwd/functions/log_inv_logit.hpp>`  
`#include <stan/agrad/fwd/functions/log_rising_factorial.hpp>`  
`#include <stan/agrad/fwd/functions/log_sum_exp.hpp>`  
`#include <stan/agrad/fwd/functions/logit.hpp>`  
`#include <stan/agrad/fwd/functions/modified_bessel_first_kind.hpp>`  
`#include <stan/agrad/fwd/functions/modified_bessel_second_kind.hpp>`  
`#include <stan/agrad/fwd/functions/multiply_log.hpp>`  
`#include <stan/agrad/fwd/functions/owens_t.hpp>`  
`#include <stan/agrad/fwd/functions/pow.hpp>`  
`#include <stan/agrad/fwd/functions/primitive_value.hpp>`  
`#include <stan/agrad/fwd/functions/rising_factorial.hpp>`  
`#include <stan/agrad/fwd/functions/round.hpp>`  
`#include <stan/agrad/fwd/functions/sin.hpp>`  
`#include <stan/agrad/fwd/functions/sinh.hpp>`  
`#include <stan/agrad/fwd/functions/sqrt.hpp>`  
`#include <stan/agrad/fwd/functions/square.hpp>`  
`#include <stan/agrad/fwd/functions/tan.hpp>`  
`#include <stan/agrad/fwd/functions/tanh.hpp>`  
`#include <stan/agrad/fwd/functions/tgamma.hpp>`  
`#include <stan/agrad/fwd/functions/trunc.hpp>`  
`#include <stan/agrad/fwd/functions/value_of.hpp>`

Go to the source code of this file.


---

[ Stan Home Page ]

*© 2011–2014,
Stan Development Team.*
